# Supplementary material for: Pathways to antibiotics in Bangladesh: A qualitative study investigating how and when households access medicine including antibiotics for humans or animals when they are ill
Source: PLoS One. 2019 Nov 22;14(11):e0225270. doi: 10.1371/journal.pone.0225270 (PMC6874326; doi:10.1371/journal.pone.0225270)
Supplement: S1 Table — (DOCX) [file pone.0225270.s002.docx]

Supplementary Table 1: Characteristics of all participating households (HH)

| Identifier^[[1]](#footnote-1)^ | **Healthcare decision maker (HDM)/ Caregiver (CG)** | **Income bracket (in 1,000 Taka)** | **Household Structure** | **Under 5s in the HH** | **Older adults in HH** | **Housing Materials** | **Ethnicity** | **Livestock in the home** |
| --- | --- | --- | --- | --- | --- | --- | --- | --- |
| RM101^*^ | HDM | <15 | Child-3, Husband, Wife and Mother | Yes | Yes | Tinshed Bari | Bengali | Yes |
| RF102 | CG | <15 | Child-2, Children's mother, Father-in-law | No | Yes | Tinshed Bari | Bengali | Yes |
| RM103 | HDM | <15 | Child-1, Son, Daughter-in-law, Husband, Wife | Yes | No | Tinshed Bari | Bengali | Yes |
| RM104 | HDM | >15 | Child-1, Hus, Wife | Yes | No | Tinshed Bari | Bengali | Yes |
| RF105 | CG | >15 | Child-1, Husband, Wife, Son, Daughter and son-in-law | Yes | No | Semi-pukka | Bengali | Yes |
| **RF106**^**^ | **CG** | >15 | **Child-1, Husband, Wife, Son and Daughter-in-law, Grandson** | Yes | Yes | **Semi-pukka** | **Bengali** | **Yes** |
| RF107 | CG | <15 | Child-1, Husband, Wife, Daughter | Yes | No | Tinshed Bari | Bengali | Yes |
| **RF108** | **CG** | >15 | **Child-2, Husband, Wife, Father & Mother-in-law, Grandmother** | **No** | **Yes** | **Tinshed Bari** | **Bengali** | **Yes** |
| **RM201** | **HDM** | >15 | **Child (Grandson)-1, Hus, Wife, Father & Mother, Son and daughter-in-law** | **Yes** | **Yes** | **Semi-pukka** | **Bengali** | **Yes** |
| RF202 | HDM | >15 | 1-Child, Children's Mother, Father-in-law, Mother-in-law | Yes | No | Tinshed Bari | Bengali | Yes |
| RF203 | CG | >15 | Husband, Wife, Aunt | No | Yes | Tinshed Bari | Bengali | Yes |
| RF204 | CG | <15 | Child-1,Husband, Wife, Sister in Law, Mother-in-law | Yes | No | Tinshed Bari | Kuch | Yes |
| **RM205** | **HDM** | <15 | **Child-1, Husband, Wife, Mother, Son** | Yes | Yes | **Mattir ghor (Mud and tin)** | **Barmon** | **Yes** |
| RF206 | HDM | <15 | Child-2,Mother, Husband, Wife | Yes | No | Tinshed Bari | Bengali | Yes |
| RF207 | CG | >15 | Child-2, Husband, Wife, Father & Mother-in-law | Yes | No | Tinshed Bari | Bengali | Yes |
| RF208 | CG | >15 | Child-3, Husband, Wife | Yes | No | Semi-pukka | Bengali | Yes |
| **RF301** | **HDM** | >15 | **Child-1, Children's mother, Mother-in-law** | Yes | **No** | **Semi-pukka** | **Bengali** | **Yes** |
| RF302 | HDM | >15 | Child-2, Children's Mother, Mother-in-law | Yes | Yes | Tinshed Bari | Bengali | Yes |
| RF303 | CG | <15 | Child-1, Hus, Wife, Daughter-in-law, Mother-in-law, father-in-law | Yes | No | Tinshed Bari | Bengali | Yes |
| RM304 | HDM | >15 | Child-2, Husband, Wife, Brother-2, Brother-in-law-2, Nephew-2, Mother. | Yes | Yes | Building, Mud house and Tinshed Bari | Barmon | Yes |
| RF305 | CG | >15 | Child-1, Husband, Wife, Brother-in-law, Mother-in-law | No | Yes | Mud House and Tinshed Bari | Kuch | Yes |
| **RF306** | **CG** | <15 | **Husband, Wife, Grandchild-2** | **No** | Yes | **Half wall and Tinshed Bari** | **Bengali** | **Yes** |
| RF307 | HDM | <15 | Child-2, Children's Mother, Mother-in-law, | Yes | Yes | Tinshed Bari | Bengali | Yes |
| RM308 | HDM | <15 | Child-1, Husband, Wife | Yes | No | Tinshed Bari | Bengali | Yes |
| **UM101** | **HDM** | <20 | **Child-1, Husband , Wife** | Yes | **No** | **Semi-pukka** | **Bengali** | **No** |
| UF102 | CG | <20 | Child-3,Husband and Wife | Yes | No | Tinshed building | Bengali | No |
| **UM103** | **HDM** | >20 | **Child-1; Father & Mother, Son, Grandmother** | Yes | **No** | **Semi-pukka** | **Bengali** | **No** |
| UF104 | HDM | <20 | Child-1, Husband, Wife, Son & Daughter-in-law | Yes | Yes | Tinshed paka bari | Bengali | No |
| UM105 | HDM | <20 | Husband , Wife, Son | No | Yes | Building | Bengali | No |
| **UM106** | **HDM** | >20 | **Husband , Wife, Father-Mother, Son & Daughter** | **No** | Yes | **Building** | **Bengali** | **No** |
| UF107 | CG | <20 | Husband, Wife, Mother-in-law, Brother-in-law and sister-in-law | No | Yes | Tinshed building | Bengali | No |
| **UM201** | **HDM** | <20 | **Child-2 (Twin), Hus (Res.*) and Wife** | Yes | **No** | **Semi-pukka** | **Bengali** | **No** |
| **UF202** | **CG** | >20 | **Child-3, Hus and Wife, Mother-in-law** | Yes | Yes | **Tin Sheet** | **Bengali** | **Yes** |
| UM203 | HDM | >20 | Husband and Wife, Son & Daughter-in-law, Grand Son & Grand Daughter-2, Maidservant | No | Yes | Building | Bengali | No |
| UF204 | CG | <20 | Grandchild-1, Mother and Son | Yes | No | Building | Bengali | No |
| UM205 | HDM | >20 | Child-1, Husband, Wife, Brother & Sister | Yes | No | Semi-pukka | Bengali | No |
| UF206 | CG | >20 | Child-3, Husband and Wife, Father | Yes | No | Semi-pukka | Bengali | No |
| UF207 | CG | <20 | Husband and Wife, Daughter | No | Yes | Semi-pukka | Bengali | No |
| UF208 | HDM | <20 | Child-1, Husband and Wife, Son & Daughter-in-law | Yes | No | Semi-pukka building | Bengali | No |
| **UF301** | **CG** | <20 | **Child-1, Mother, Mother-in-law, Father-in-law** | Yes | Yes | **Building** | **Bengali** | **No** |
| **UF302** | **CG** | >20 | **Child-1, Husband, Wife, Mother-in-law, Father-in-law, Brother-in-law-2, Niece** | Yes | Yes | **Building** | **Bengali** | **No** |
| UF303 | HDM | <20 | Child-2, Husband, Wife | Yes | No | Tinshed building | Bengali | No |
| UF304 | CG | <20 | Child-1, Wife, Husband | Yes | No | Building | Bengali | No |
| UF305 | HDM | >20 | Child-1, Wife, Husband, Father-in-law | Yes | Yes | Building | Bengali | No |
| UF306 | CG | >20 | Husband, Wife, Son-3, Mother-in-law | No | Yes | Building | Bengali | No |
| **UF307** | **HDM** | >20 | **Husband, Wife, Daughter-2** | Yes | **No** | **Building** | **Bengali** | **No** |
| UF308 | CG | >20 | Husband, Wife, Daughter, Son, Nephew | Yes | No | Building | Bengali | No |
| **UF309** | **CG** | >20 | **Husband, Wife, Son-2, Daughter, Daughter-in-law, Grandchild-2** | Yes | **No** | **Building** | **Bengali** | **Yes** |

^*^R=rural and U=urban area, F=female and M=male participants.

^**^Households with an ill family member and follow up interview are shown in bold

1. Identifiers are study identifiers and match our published data, but are therefore not consecutive. [↑](#footnote-ref-1)
