# Supplementary material for: Pathways to antibiotics in Bangladesh: A qualitative study investigating how and when households access medicine including antibiotics for humans or animals when they are ill
Source: PLoS One. 2019 Nov 22;14(11):e0225270. doi: 10.1371/journal.pone.0225270 (PMC6874326; doi:10.1371/journal.pone.0225270)
Supplement: Attachment — Submitted filename: Response to reviewers.docx [file pone.0225270.s003.docx]

| Number | Editorial comments | Author response |
| --- | --- | --- |
| 1. | Please ensure that your manuscript meets PLOS ONE's style requirements, including those for file naming. The PLOS ONE style templates can be found at <http://www.journals.plos.org/plosone/s/file?id=wjVg/PLOSOne_formatting_sample_main_body.pdf> and <http://www.journals.plos.org/plosone/s/file?id=ba62/PLOSOne_formatting_sample_title_authors_affiliations.pdf> | Supporting information has been and saved using file naming requirements. |
| 2. | Please include copies of the interview guide(s) used in the study, in both the original language and English, as Supporting Information, or include a citation if they have been published previously. | Interview guides in the original language (Bangla) and English are available at: <https://doi.org/10.17028/rd.lboro.9333158.v1>  We have amended the manuscript including this information at line 134. |
| 3. | We note that you have stated that you will provide repository information for your data at acceptance. Should your manuscript be accepted for publication, we will hold it until you provide the relevant accession numbers or DOIs necessary to access your data. If you wish to make changes to your Data Availability statement, please describe these changes in your cover letter and we will update your Data Availability statement to reflect the information you provide. | Original data from the study can be accessed via the Loughborough University data repository at: <https://doi.org/10.17028/rd.lboro.8953118.v1> |
| 4. | Please include captions for your Supporting Information files at the end of your manuscript, and update any in-text citations to match accordingly. Please see our Supporting Information guidelines for more information: <http://journals.plos.org/plosone/s/supporting-information>. | Added and in-text citations changed to match accordingly |
|  | Reviewer 1 |  |
| 5. | Reviewer #1: The article is interesting as it was conducted qualitatively so that it can explore the depth of problems related to acquisition of medicines and antibiotics among low income households. However, there are some notes to improve the clarity of the article. | Our thanks for this encouraging comments, we are glad to address specific comments. |
| 6. | First, the title does not really reflect the contents. The pathways to antibiotics are not clear, as stated in line 463-6. It is suggested to include the term "medicines". So that, the title becomes something like "Pathways to medicines including antibiotics in Bangladesh: ...". Short title should also be changed with "... households in Bangladesh" or "...Bangladesh household" | We agree that we have information about pathways to medicines in general, but we did focus questions on antibiotics were we could. We have changed our title to:  Pathways to antibiotics in Bangladesh: A qualitative study investigating how and when households access medicines including antibiotics for humans or animals when they are ill  Short title: Pathways to antibiotics for households Bangladesh |
| 7. | Inconsistencies in writing quotes:  Some quotes end with fullstop, but some do not and even have no unquote, such as shown in line 216, 232, 280, 334. Some quotes put fullstop after unquote such as line 325, 328.  Some quotes have their respondent ID in brackets, while most do not, such as shown in line 391, 398, 401. | We have reviewed all for consistency, and our tracked changes manuscript shows amendments. |
| 8. | It seems that the article was prepared in hurry because some references are still blank or not formatted in Plos ONE Vancouver style. The cases are shown in line 58,521, 522, 544, 568. | Our apologies, we processed references through EndNote and didn’t sufficiently check that these had been correctly formatted. We have checked and corrected all by hand. |
| 9. | Table 1 misses some lines | We are not sure what lines are missing. The identifying numbers are not consecutive, and we have added a note to this effect in case this was confusing. We choose not to change our study identifiers as this allows matching to our published data sources. |
| 10. | References should be written in a correct, consistent manner. The journal names vary in style. Some are written in Title Case, such as reference 25; most are in Sentence case; and some others are unclear such as referencce 4, 17, 19. It's not necessarily to write the place of Lancet publisher (reference 11). Reference 1, 5, 6, and 7 are not written consistently. In addition, reference number 6 should be deleted or revised as the link is 404 (Page not found). | Our apologies, we processed references through EndNote and didn’t sufficiently check that these had been correctly formatted. We have checked and corrected all by hand. |
| 11. | It is commonly preferable to write numbers <=10 as a word. For example, 7 is written as seven. The cases are in line 37, 157, 182, 253, 265. | We have amended as requested, please see track change version. |
| 12. | It is also favourable to avoid the use of first-person pronouns, i.e. "we". | In qualitative research, it is quite common to use first-person pronouns. We prefer to keep to first-person unless the editorial team also prefer we remove these. |
| 13. | Third, in the beginning of the article the authors deligently write the full form of abbreviations in their first mention, but when in the middle part the authors forget to mention the full form of MBBS (line 193); TB (line 286); and icddr,b (line 287). Could MBBS be explained in more details? | We have attended to each of these, please see track change version. |
| 14. | Fourth, what is the justification to use only poor households. Do middle-income households may have different pathways? Both groups may have similar preference if offered with low prices and easy access of drugs. | We have amended our text to be clearer that we included not only the lowest income households, but two strata at the lower end of the income distribution. We now state that:  “Household income cut-offs were based on rural and urban mid-range estimates of household income adjusted for inflation {Bangladesh Bureau of Statistics, 2011 #163}, focusing our research on two strata at the lower end of the income distribution.” Line 109-111  We have also reviewed language throughout to be consistent with this. We believe it is sensible to focus on lower income households whose choices are more restricted, but agree with the reviewer that if we had focussed on only the poorest our findings would be limited. |
| 15. | Fifth, in line 109 the authors mention the intention to include minority indigenous population. What criteria were used to ensure that the respondent is an indigenous person? Should they have indigenous maternal and paternal grandparents? What if a potential respondent has inter-racial or interethnic parents? WHat kind of ethnicities is dwelling in the research area? | Ethnicity was self-declared by participants, and we have added this information to our methods section:  “Households were recruited and interviewed by local researchers who visited house to house using a screening questionnaire which include questions about household composition, income and ethnic group.” (line 124/5) |
| 16. | Sixth, in line 119 are all members of the team able to communicate in English? Please provide more details. | We have amended our description of our use of translation to include this information. We now state:  “All members of the qualitative analysis team except PL were bilingual in Bangla and English.” |
| 17. | Seventh, was the interview guide developed bilingual in English and Bangla? Please provide more details. | We have added links to both version of the interview guide, please see also response 2 above. |
| 18. | Eighth, in line 130 how did the researchers explain the concepts of antibiotics, side effects, and antimicrobial resistance during the interview? How could the respondents differ antibiotics from other medicines or even vitamin and supplements? The information is better being written in methods section rather than in results. | We have added additional details, along with links to our interview guides. We now state:  “The terms antibiotics and AR were explained where needed by field staff. We began by using these terms and asking if they were known, but where people did not know the terms they explained further using common language (see interview guide), provided examples of antibiotic names and packaging, provided leaflets published by icddr,b and asked to see any medicines in the home to identify any antibiotics currently being used.”  See lines 138+ |
| 19. | Ninth, in line 136 why were only a third of recorded interviews translated into English? What criteria were used to select the records to be translated? | We have added additional information about our translation process, see response 29 below. |
| 20. | Tenth, what is the differences between drug shops and community pharmacies in the study? The reference 12 mentions "private pharmacies" instead of "drug shops". Are they similar? (line 70-8) | We only use the term ‘drug shop’ as the commonly used generic name for the range of places where medicines can be directly purchased by the public.  We agree that other terms can be confusing, which is why we choose to use only this one in keeping with other literature in the field (see our references 15, the SIAPS study in particular).  We have added an additional sentence to our results section, citing the SIAPS report:  “Drug shops are the common name for retail locations which sell allopathic medicines, regardless whether they are licensed (15).” |
| 21. | Eleventh, in line 525-6 the authors wrote "Eliminating direct access through 525 drug shops would not impact on this, and regulating to allow antibiotic access only via prescriptions would not necessarily improve practice." How could the authors create these claims? What evidence can be given? Some published articles about self-medication with antibiotics in developing countries can be incorporated in the discussion section. | We have reworked our discussion in response to this and other comments (22, 38, 89).  See lines 554-609 |
| 22. | Lastly, in line 535 the authors wrote "...lack of access to qualified doctors will push people into the black market." What does the term "black market" here mean? What evidence can be given? | We have reworked our discussion in response to this and other comments (21, 38, 89).  See lines 554-609 |
|  | Reviewer #2 |  |
| 23. | Thank for this informative research that is highly needed. | Thank you for this encouraging comment. |
| 24. | Abstract: I am not sure 'loose economic and regulatory system' is the correct term? There is regulation in Bangladesh, including the newly implemented 'model pharmacies' which promote prudent use of antibiotics. The issue is that the regulation is not enforced correctly.  In the methods of the abstract you should state the sampling method used to identify participants and why how saturation of data was achieved.  In the findings, rather than reporting 8 out of 13, perhaps it would be more appropriate as per chosen methods to discuss that antibiotics were reported to be one of the common therapies chosen for illness or words to that effect. It would be valuable to know if the households who opted for antibiotics were the more wealthy or this behaviour was reported across the different income categories.  The last sentence of the conclusion is important - as often access is so precarious and should not be restricted easily in order to tackle AMR. Would the authors please write this sentence more clearly, highlighting this issue in efforts to tackle AMR in LMICs. | We have amended to state “loose regulation and enforcement”.  Within the 300 word limit we have not been able to add this useful additional information.  Again, we are limited to what we can include in the abstract given 300 word limit (currently 292 words). We have included these details in the main body of the text, but were unable to add more detail without sacrificing clarity in this very brief summary. We have amended to state instead ‘most’ instead of ‘8’ households.  We have amended the conclusion, although again we are restricted by word count. The conclusion now reads:  “In Bangladesh, unregulated drug shops provide an essential route to medicines including those prescribed in the formal sector. Wherever licensed suppliers are scarce and expensive, regulations which prohibit this supply risk removing access entirely for many people.”  We hope this is clearer, and alludes to similar issues in other countries. |
| 25. | Main manuscript: Line 57 - sentence can be reworded, not sure impacts used in plural sense reads well | We have amended to read:  “Low and middle income countries (LMICs) already experience the worst impact” |
| 26. | Line 58 - there is a plethora a papers which can be used for reference including the Lancet series on AMR and papers my Laxminarayan et al | Our apologies for this error. We have re-inserted our references here. |
| 27. | Line 62 - and duration ...  line 73 Bangladeshis not Bangladeshi's  Line 80 Furthermore,.... | Corrected, please see track change version. |
| 28. | Line 102 - Mirzapur district has an extensive surveillance programme for childhood infectious diseases that reaches thousands of households via the Child Health Research Foundation - See ANISA study in the Lancet | We are aware that there have been research and surveillance studies in Mirzapur district conducted by both icddr,b and the Child Health Research Foundation. There are 14 unions (unitary areas) in Mirzapur district, and 8 Unions have been involved in past research or surveillance studies. We excluded these 8 unions from the PAUSE study. For the PAUSE study we selected households from the remaining 6 unions where previous studies had not been conducted. Hence the study area households have not been in previous research studies.  To aid clarity, we have now state:  “we selected one urban area (Tongi, within Gazipur District) and one rural area (within Mirzapur District)” |
| 29. | methods So if only 1/3 of transcripts were translated is the data presented in this manuscript only from that batch or a wider selection was presented and translated for the purpose of the paper? | We considered our approach to translation carefully, and included a fuller description of the process in our protocol paper. We translated 1/3 of interviews in full, the remainder were transcribed and read in Bangla, but the summary data (including quotes) were translated into English by the researchers. We therefore used the whole set of interviews for this research. We have amended our text to make this process clearer:  Line 148 onwards “We considered our approach to translation carefully (ref to protocol) and used an approach which kept first stage analysis in Bangla in most cases. A third of interviews were translated in full and these were used to develop our analysis plan. The field team were asked to identify rich interviews from both rural and urban areas for this purpose. For those interviews not translated in full, the bilingual research team members coded Bangla transcripts into framework charts, and these charts were then translated for sharing with the full team for further analysis. As analysis progressed, we checked the translated material against the Bangla transcripts where needed”  And we have made some additional small edits to the analysis description to ensure it is clear that all interviews were used in analysis. |
| 30. | Did you do analysis of data as you gathered it? Did that also inform the saturation? | We have moved and amended the sentence mentioning data saturation from results to methods. It now reads:  “We continued recruitment until our data saturation was reached, which we judged against our sample targets and in discussion with the field researchers to establish when they felt they were no longer hearing no material.” |
| 31. | The first two sentences of results belong in the methods section | See response 30 above, we have moved the first sentence to the methods section, we agree this was an error.  We would prefer to keep the description of recruitment rate in the results section. Although it is more common to include this material in methods of a qualitative paper, this is a stylistic choice and we believe that the readers of this journal will be more used to reading all details of response rate and sample description in the results section.  We can amend this is the reviewer and/or editor disagree with our choice. |
| 32. | The drop out section is a bit confusing - can you break it down in urban vs rural and define the dropout for each? | We have amended this text as requested, and it now reads:  “We approached a total of 49 rural and 67 urban households. In the urban sites, we struggled to find households with animals and therefore we relaxed this criterion. Of the 67 households  approached, 36 (54%) households did not meet inclusion criteria. Of the 31 eligible households, seven (22.5%) refused to participate and we interviewed the remaining 24 households (77.5% of those eligible, 36% of those approached). In the rural site, of the 48 households approached 22 (46%) did not meet inclusion criteria, and in two households neither the decision maker nor carer were at home. We interviewed the remaining 24 households (100% of those eligible, and 50% of those approached” |
| 33. | It would help to provide the average income in professional urban and rural populations for Bangladesh, the difference between these two categories doesn't appear to be great | As described in response 14 we have amended the manuscript to be more explicit about this cut off. We hope this is now clearer. |
| 34. | Line 183 - did you explore if there were specific conditions that they would seek help from 'fakirs' e.g. did infections feature in this? Or were there specific conditions they treat? It would be interesting in terms of delayed therapies for infections etc. | We did not explore this any further, as the interviews were already long as this was not the focus of this research. We are reluctant to say much more on the basis of limited data here, but agree that role of traditional healers is interesting. |
| 35. | Line 265 - again i would refrain from giving numbers as in the context of the methods selected in this study they don't add anything. Better to focus on the findings and what they mean. | We have removed the number here. |
| 36. | Did you explore whether the medicines for human use were ever given to the animals? Antibiotics specifically? | Yes we did ask about this, and as reported to us this was a rare occurrence. We have added a new paragraph to say:  “We also asked households about use of human medicines for their animals. Only one participant reported this practice. One rural woman (RF305) reported giving "napa” (paracetamol/acetaminophen) which she also called "fever medicine” mixed into rice or water to her chickens when they were sick.”  Line 433+ |
| 37. | It would be useful and important to share the translated interview guide that was used to conduct the interviews - particularly when reading the section about knowledge and understanding under the pathways to antibiotics section of the results. | Please see response 2, we have now done this. |
| 38. | It would be good if in the discussion you can refer to the issue of access to antibiotics via informal routes, as described by some of your participants and the consequences of further restrictive regulation of this for poor households using some of the cases that you have followed up and provide details of in table 2. This is a critical point in the global efforts to tackle AMR, specifically in LMICs. | We have reworked our discussion in response to this and other comments (21, 22, 89).  See lines 554-609 |
| 39. | The sentence beginning with 'Qualified doctors may be...' in your conclusions needs refining - it is not clear who relies on drug sellers. The conclusion could be written more tightly. The last two sentences particularly. The last sentence - there are examples of this in Africa - the ADDOs (accredited drug dispensing outlets) - it would be good if this is referred to in the discussion.  Also worth mentioning the model pharmacies in Bangladesh and the role these may have going forward - it is briefly alluded to but not explained in detail. The pharmacy of Kumudini hospital in Mirzapur for example is a model pharmacy. | We have reworked our discussion and conclusions in response to this and other comments (21, 22, 38).  We now include more information about model pharmacies (lines 603-607) and mention the ADDO model from Africa (Lines 607-609).  The Kumudini pharmacy only joined the model pharmacy initiative during our data collection period (June 2017), so we do not believe it will have changed the experiences of participants. However, we added reference to this initiative in our discussion (lines 605) |
|  | Reviewer #3: Lucas et al present a qualitative assessment of access to antibiotics for human and animal use in Bangladesh. This is an interesting and thorough assessment of an important problem. It focuses on the two major problems relating to antibiotics--overuse and lack of access with a one health type approach. | Thank you for this positive comment. |
| 40. | I had a few minor comments: introduction line 58 (ref) mentioned--add a reference Line 71 replace dash with "to" in "eighty-ninety" Line 80 what does "highly plural health provision" mean? Table 1 has funny formatting of lines--carefully edit Left upper quadrant Discussion line 568--again "(ref)" needs to be corrected Line 574 "For humans" can be removed | We have amended all, please see track changes version. |
